# Rapid bedside measurement of reactive oxygen species in neonates: a pilot study

**DOI:** 10.3389/fped.2025.1611197

**Published:** 2025-10-08

**Authors:** Yuta Iijima, Mamiko Endo, Tadashi Shiohama, Yoshiteru Osone, Kimiko Kazumura, Naoki Shimojo, Hiromichi Hamada

**Affiliations:** ^1^ Department of Pediatrics, Graduate School of Medicine, Chiba University, Chiba, Japan; ^2^ Perinatal Medical Center, Chiba University Hospital, Chiba, Japan; ^3^ Department of Pediatrics, International University of Health and Welfare Narita Hospital, Narita, Chiba, Japan; ^4^ Global Strategic Challenge Center, Hamamatsu Photonics K.K., Hamamatsu, Shizuoka, Japan; ^5^ Center for Preventive Medical Sciences, Chiba University, Chiba, Japan

**Keywords:** reactive oxygen species, hypochlorite ions, neonatal intensive care unit, bedside measurement, biomarker

## Abstract

**Introduction:**

Biomarkers for the early detection of severe neonatal conditions, such as necrotizing enterocolitis and sepsis, remain inadequate. Reactive oxygen species (ROS) produced during neutrophil activation are emerging as potential biomarkers of these diseases. This study aimed to evaluate the feasibility of bedside ROS measurement and establish baseline levels in neonates.

**Methods:**

Using the FLP-H4200 fluorescence-based system, OCl^−^ were measured from 3 μl of whole blood. Twenty neonates (13 preterm and seven full-term) were included. On postpartum day 4, OCl^−^ levels were measured using residual blood samples.

**Results:**

Baseline OCl^−^ levels averaged 31,340 ± 10,674 and 26,022 ± 11,363 in full-term and preterm neonates, respectively (*p* = 0.35). No significant correlations were observed between OCl^−^ levels and gestational age, birth weight, maternal milk intake, bilirubin, and C-reactive protein levels.

**Discussion:**

The FLP-H4200 system is feasible for rapid and minimally invasive ROS measurement in neonates. Although no significant associations with clinical factors were identified, elevated ROS levels compared with those in adults suggest neonatal adaptation to oxidative stress. Further research is required to evaluate ROS dynamics progressively and their clinical use in neonatal disease prediction.

## 1 Introduction

The absence of reliable biomarkers in neonatal medicine complicates the early diagnosis of life-threatening conditions, such as necrotizing enterocolitis (NEC) and sepsis, which have high mortality rates in neonatal intensive care units (NICU) (1–3). Early detection and treatment could reduce mortality and ensure a good prognosis. Although C-reactive protein (CRP) reflects the inflammatory response, it lacks sensitivity and specificity for early detection, as its levels gradually rise only after disease progression (2, 3). The mortality rate of NEC and sepsis is high; therefore, CRP levels is not a sensitive marker for detecting serious diseases in the early phase. Leukopenia, leukocyte increase, and neutrophil count are used to diagnose sepsis, but white blood cell counts are influenced by gestational age, maternal disease, and the presence of fetal diseases. Hence, biomarkers for early detection have been explored; however, no markers that can be routinely measured in NICU clinical practice have been established. Several proteins (amyloid A, IL6, and TNFα) are being considered. However, they have not been used practically owing to problems with sensitivity and specificity, time required for measurement, and cost (4).

Neutrophil activation is deeply involved in the pathogenesis of these inflammatory diseases (3–7). Reactive oxygen species (ROS), such as O_2_^−^ •, H_2_O_2_, hydroxyl radicals, and OCl^−^, are produced during neutrophil activation. Recently, we developed a technique to measure ROS using only 3 µl of whole blood (8) and simultaneously measures chemiluminescence and fluorescence, indicating O_2_^−^ • and OCl^−^ generation of neutrophils stimulated by agonist-stimulated neutrophils. Using this method, we reported that ROS levels were elevated in several adult infectious diseases, preceded by elevated CRP levels, and clarified the relationship between ROS and various adult diseases (8–13). These data suggest that ROS levels may predict various acute inflammatory diseases. Currently, there have been no reports on the feasibility of measuring ROS levels at the bedside in the NICU. This study aimed to establish a standard method for measuring ROS levels in neonates using a neutrophil activity evaluation system.

## 2 Methods

### 2.1 Participants and blood sampling

This study included neonates born at the Chiba University Hospital between June 2023 and March 2024. We approached a total of 20 mothers to participate in this study and all provided written informed consent (consent rate: 100%). The exclusion criteria were congenital (neurological disorders, metabolic disorders, and chromosomal disorders) and infectious diseases (chorioamnionitis, congenital infections, and sepsis). Preterm neonates (<37 weeks old) were admitted to the NICU, whereas full-term neonates (37–41 weeks) remained in the maternity ward. In neonates admitted to the NICU, OCl^−^ was measured using the remaining blood samples between days 3 and 5 of life. Samples were collected using heparinized capillaries, stored at room temperature, and measured within one hour after collection. In our previous study, reproducibility was confirmed within 4 h (CV <a few %); in this study, stricter conditions were applied. For neonates who did not require NICU hospitalization, OCl^−^ levels were measured using the remaining blood samples drawn for neonatal mass screening on day 4 of life. We analyzed the amount of breast milk, bilirubin, and CRP levels and the average SpO_2_ on the day of blood collection as potential covariates for the OCl^−^ values.

### 2.2 Measurement of ROS

We used a newly developed popularized model (FLP-H4200, Hamamatsu Photonics K.K., Hamamatsu, Japan) that could only monitor OCl^−^ (12). Briefly, 3 µl of whole blood was mixed with 750 µl of incubated Ringer–Hepes buffer (RH buffer: 154 mM NaCl, 5.6 mM KCl, 10 mM Hepes, pH 7.4), containing 1 mM CaCl_2_ and 10 µM aminophenyl fluorescein (APF; GORYO Chemical, Sapporo, Japan), which is as fluorescence reagent of OCl^−^, in a dedicated fluidic chip. The fluorescence signals were monitored for 1,500 s. The reaction solutions were stimulated with 0.1 µM phorbol 12-myristate 13-acetate (PMA; Sigma-Aldrich) for 150 s after starting the measurement. The increased fluorescence signal intensities after PMA stimulation were calculated using dedicated analysis software and used as evaluation values. Details have been described previously (8, 12). The measurement protocols and workflow were described in detail in a previous study (8). According to that study, sample preparation and setup took about 7 min, and the measurement process took a further 25 min. The specificity of the APF-based fluorescence signal for hypochlorous acid ions (OCl^−^) has been verified previously using this system (14).

### 2.3 Calculation of the maternal milk use ratio

We calculated the maternal milk use ratio by dividing the maternal milk use by the total amount of milk and multiplying the result by 100. We classified a maternal milk ratio of >70%, 30%–70%, and <30% into the maternal milk, mixed, and artificial milk groups, respectively.

### 2.4 Statistical analysis

Data were analyzed using GraphPad Prism version 10 (GraphPad Software, San Diego, CA, USA) and R version 4.4.0. Analyses were performed using student's *t*-test, Welch's *t*-test, one-way ANOVA, and Pearson's correlation analysis. Statistical significance was set at *p* < 0.05.

### 2.5 Ethical considerations

All research involving human participants was conducted in accordance with the Declaration of Helsinki and relevant regulations. This study was approved by the Ethics Committee of Chiba University Hospital (Approval Number: #5-167). The research content was explained to the patient's family, and written consent was obtained prior to their participation.

## 3 Results

### 3.1 Participant characteristics

Written informed consent was obtained from the parents of 20 neonates (seven full-term and 13 preterm). The mean ± standard deviation measurement date was 4.0 ± 0 and 4.0 ± 0.8 days for the term and preterm groups, respectively. OCl^−^ was tested using residual samples from routine testing. Blood sampling was not required in any of the cases (Table 1).

**Table 1:** Characteristics of participants.

Characteristic	All (*n* = 20)		Full-term (*n* = 7)		Preterm (*n* = 13)		*p*-value
Gestational age (weeks), mean (SD)	35.3	(2.7)	38	(0.6)	33.8	(2.2)	<0.001
Birth weight (g), mean (SD)	2,145.2	(667.9)	2,810.1	(375.2)	1,787.2	(525.7)	<0.001
Male, no. (%)	15	(0.75)	4	(57)	11	(85)	0.3
Hospitalization, no. (%)	13	(0.65)	0	(0)	13	(100)	<0.001
Age of days at measuring the OCl^−^, mean (SD)	4	(0.63)	4	(0.0)	4	(0.8)	>0.9
Repeat blood sampling, No. (%)	0	(0)	0	(0)	0	(0)	>0.9

No, number; SD, standard deviation.

**Figure 1:**
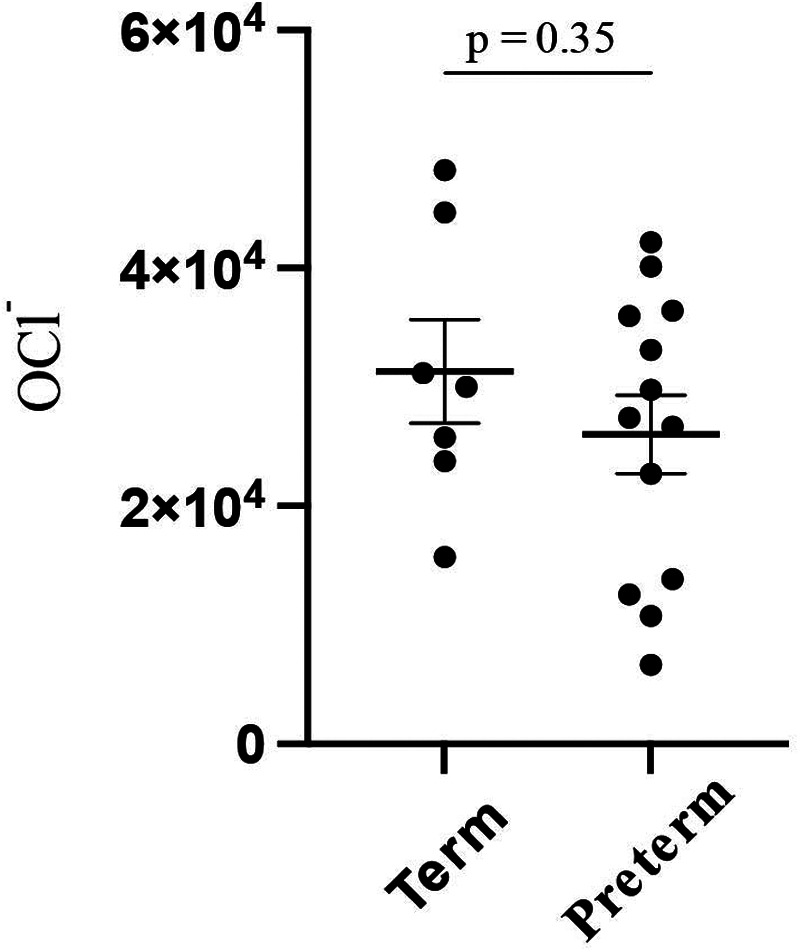
The OCl− levels of whole blood of full-term and preterm neonates.

The overall OCl^−^ level was 27,900 (±11,700), whereas it was 31,300 (±10,700) and 26,000 (±11,300) in full-term and preterm infants, respectively, with no significant difference (*p* = 0.35) (Figure 1).

### 3.2 Association between factors at birth and the OCl^−^ levels

**Figure 2:**
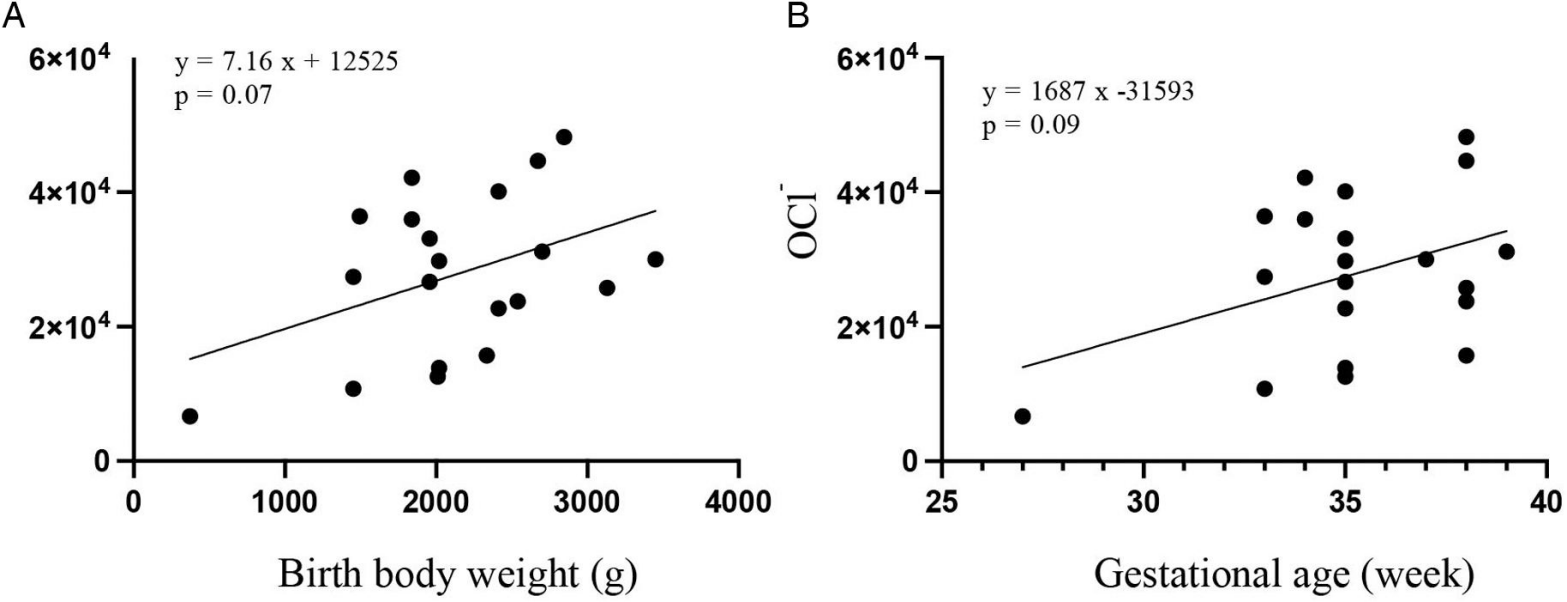
Scatter plots and regression lines between the OCl− levels and birth weight **(A)** or gestational age **(B)** by the Pearson's correlation analysis.

The correlation coefficient between birth weight and OCl^−^ levels at 4 days of age was 0.19 (*p* = 0.07) (Figure 2A). The correlation coefficient between gestational age and OCl^−^ levels at 4 days of age was 0.16 (*p* = 0.09) (Figure 2B).

### 3.3 Association between factors after birth and the OCl^−^ levels

**Figure 3:**
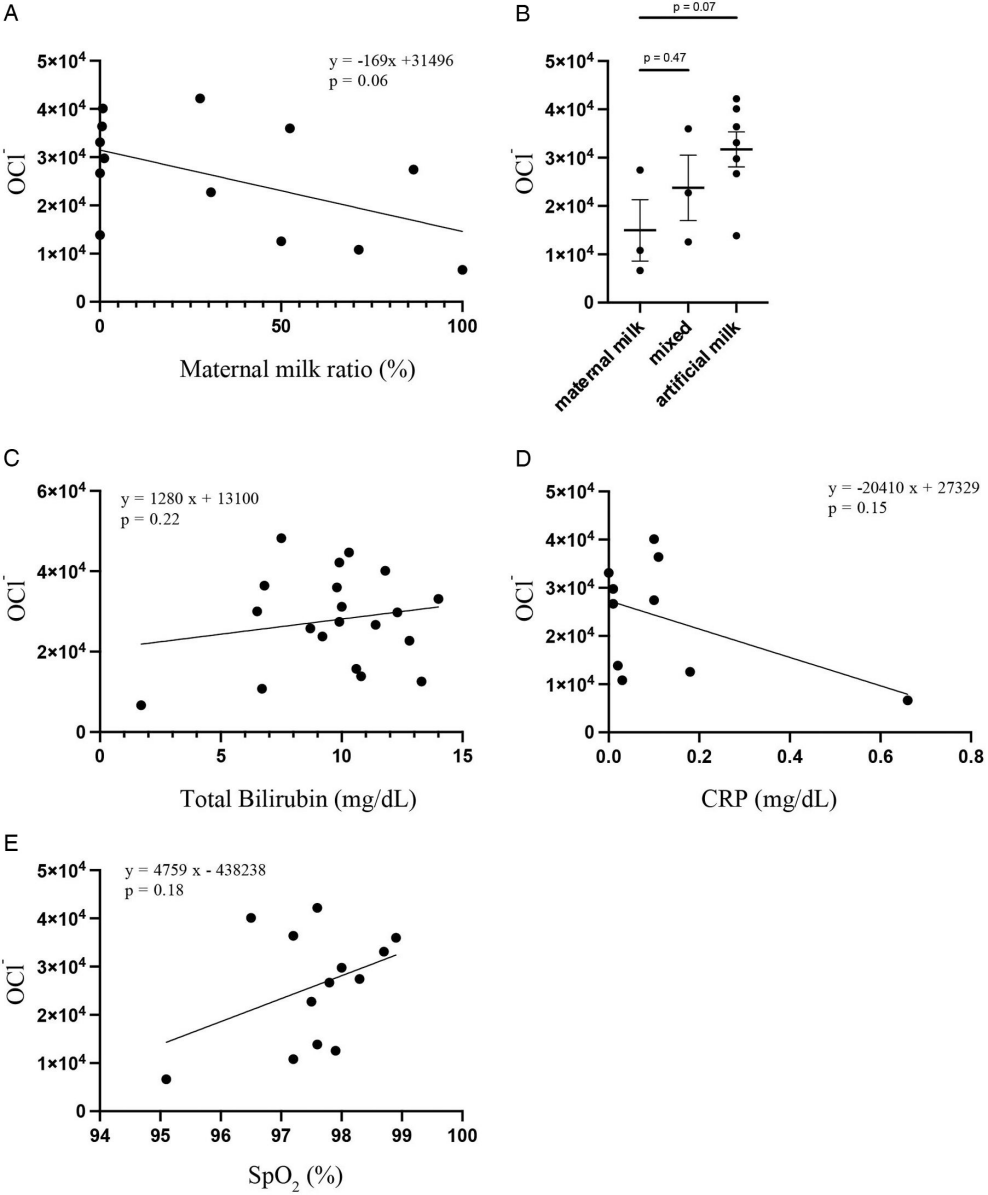
Scatter plots and regression lines between the OCl− levels and milk feeding type by Pearson's correlation analysis **(A)** OCl− levels in maternal milk (*n* = 3), mixed (*n* = 3), and artificial milk (*n* = 7) groups were compared by student's *t*-test **(B)** scatter plots and regression lines between the OCl− levels and total bilirubin **(C)**, CRP **(D)**, and SpO2 **(E)** by Pearson's correlation analysis.

The correlation coefficient between the ratio of maternal milk and OCl^−^ levels at 4 days of age was −0.27 (*p* = 0.06) (Figure 3A). There was no significant difference in the OCl^−^ levels between the maternal milk and mixed groups and maternal and artificial milk groups (maternal milk vs. mixed: *p* = 0.47; maternal milk vs. artificial milk: *p* = 0.07) (Figure 3B). No significant correlations were observed between OCl^−^ levels and clinical factors, such as bilirubin (*r* = 0.12, *p* = 0.22) (Figure 3C), CRP levels (*r* = −0.24, *p* = 0.15) (Figure 3D), and SpO_2_ (*r* = 0.14, *p* = 0.18) (Figure 3E).

## 4 Discussion

This study established a novel and minimally invasive method for ROS measurement in neonates using a fluorescence-based device. The minimal blood volume requirement of this method is particularly advantageous for reducing iatrogenic anemia. No significant associations with clinical factors were observed. We have established a reference range for OCl^−^ in adults from a large clinical study conducted with the help of 2,677 volunteers using FLP-H3200 (15). In this study, FLP-H4200 is the improved version of FLP-H3200 (12), and the fluorescence signal level obtained was approximately three times higher because of the stronger excitation light intensity (*y* = 2.9027x + 5,616). The adult mean value of the FLP-H4200 obtained by calculating the correlation coefficient between FLP-H3200 and FLP-H4200 is 20,000 (±11,700). Based on reference values obtained from a large adult cohort measured by the same device (FLP-H4200) and the same research group, neonatal OCl^−^ levels appeared higher (*p* = 0.008). However, as this was an indirect comparison, these findings should be interpreted with caution. To our knowledge, this is the first report that the OCl^−^ levels can be measured using only 3 µl in NICU. Previously, the OCl^−^ levels can be assessed using 200 µl of whole blood samples (16). However, this volume is excessively high for neonates, especially premature infants. Neonates easily develop iatrogenic anemia if blood samples are collected several times (17). Thus, 3 µl is sufficient to obtain from surplus samples used in daily examinations, such as serum bilirubin level and blood gas analysis. This is a significant advantage in neonatal medicine because additional blood sampling is unnecessary.

Births present a significant oxidative challenge due to the shift from a relatively low-oxygen intrauterine environment to a high-oxygen extrauterine environment (18). This physiological shift may contribute to elevated ROS activity in neonates. We expected the ROS levels to change according to gestational age or birth weight; however, we did not find a significant correlation between ROS levels and gestational age or birth weight. The production of antioxidants in preterm infants may differ from that in full-term infants. Further studies are required to elucidate the changes in ROS levels that accompany aging during the neonatal period.

ROS levels in healthy neonates may be affected by factors besides gestational age and birth weight. The relationship between the OCl^−^ levels and maternal milk was of interest, although not significant (*p* = 0.06). Maternal milk contains more antioxidants than artificial milk, such as glutamine, arginine, carotenoids, B-cryptoxanthin, melatonin, HMO, and lactoferrin (19, 20). Maternal milk provided by mothers of full-term and preterm infants have better antioxidant properties than formula, and preterm and full-term maternal milk have equal resistance to oxidative stress (21, 22). Bilirubin is an antioxidant; therefore, we hypothesized that higher bilirubin levels would correspond to lower OCl^−^ levels (23). However, we could not detect a significant correlation between the OCl^−^ and bilirubin levels. We found no significant correlation between the OCl^−^ and CRP levels. We expected that CRP and ROS levels would correlate positively under infectious conditions. In this study, we measured the ROS levels in a steady state. Therefore, ROS may not correlate with CRP. Excessive oxygen administration leads to the production of ROS (24). We assumed that ROS levels would change if SpO_2_ levels were low. However, we did not observe a significant correlation between the OCl- and SpO_2_ levels. This may not reflect oxygen toxicity, because there were no cases of oxygen administration. To evaluate the relationship between oxygen toxicity and the OCl- levels, assessing the oxygen dosage and SpO_2_/FiO_2_ ratio may be necessary. Most previous studies showing correlations were performed in diseased neonates or under different measurement conditions, which may explain the discrepancies with our pilot study focusing exclusively on healthy neonates.

Excessive ROS is known as oxidative stress and may lead to inflammation or multiple diseases. Oxidative stress may be important in many neonatal diseases, such as bronchopulmonary dysplasia (BPD) (25–28) or retinopathy of prematurity (ROP) (5, 29, 30). Therefore, we believe that the OCl^−^ levels help us understand many diseases and serve as a biomarker for neonatal diseases. Studies on the OCl^−^ levels measurement by this method as a biomarker for these diseases are mandatory eventually. As this was a pilot study, the findings should be regarded as preliminary and serve as a basis for future multicenter studies.

## 5 Limitations

This investigation was conducted as a pilot study, intentionally limited to disease-free neonates from a single institution. Conducting the study at a single facility also helped minimize variability due to differences in measurement environments, operator techniques, and NICU admission criteria across institutions. Therefore, some non-significant findings (e.g., Figures 1, 3B) may reflect limited statistical power due to the small sample size rather than the absence of true differences. In addition, the ROS measurement device used in this study is still being improved, so the number of patients who could be included during the study period was limited. Furthermore, we conducted a *post-hoc* power analysis and calculated the effect size (Cohen's d). In the comparison between term and preterm neonates, Cohen's d was calculated as 0.45, and *post-hoc* power analysis revealed that the power of this analysis was 15%. This indicated a small to moderate effect and the study may have been underpowered to detect a significant difference. For the comparison based on milk type, Cohen's d was calculated to be 0.76, and *post-hoc* power analysis revealed that the power of this analysis was 54.3%. This indicates that the effect size is medium to large, but it is possible that this study did not have sufficient power to detect significant differences. Additionally, our data excluded a time-course. As neonates experience dramatic changes in transitioning from a relatively low-oxygen intrauterine environment to a high-oxygen extrauterine environment, ROS levels may vary depending on the number of hours or days after birth. Future studies should measure the ROS levels progressively to account for these changes.

Furthermore, as all neonates were kept in room air for at least 1 h before blood sampling, FiO₂ could not be used as an evaluation variable in this study. Instead, we used SpO₂ as an indicator of oxygenation. However, this may not accurately reflect the oxidative stress associated with oxygen exposure. In future studies, we need to analyze FiO₂ or the SpO₂/FiO₂ ratio and the PaO₂/FiO₂ ratio in neonates receiving oxygen supplementation to more accurately assess this relationship.

This study excluded patients with certain diseases. Therefore, conclusions regarding potential disease associations could not be drawn from our data. Further investigation is required to explore these relationships.

Another limitation is that we could not directly compare our results with other established ROS detection methods, because these platforms were not available in our facility. In our previous adult studies, correlations between OCl^−^ and oxidative stress markers such as 8-OHdG, HEL, and PRL varied depending on the clinical context (9–11). This discrepancy likely reflects that conventional markers measure oxidative products generated as a consequence of ROS-mediated damage, whereas our system directly monitors ROS production itself (hypochlorite ions). Therefore, the ability of our system to detect ROS production at an early stage represents a unique advantage, particularly in the neonatal setting where timely intervention is critical.

Overall, we successfully measured ROS levels using small amounts of blood collected from the bedside in the NICU. Timely and regular measurements of ROS in neonates in the NICU may be helpful for the early diagnosis and treatment of neonatal diseases. In future studies, we intend to analyze ROS levels over time, potential confounding factors, and the relationship between ROS levels and inflammatory diseases in preterm and term infants.

## Data Availability

The original contributions presented in the study are included in the article/Supplementary Material, further inquiries can be directed to the corresponding author.
